# Registered Hospitals and Asylums

**Published:** 1913-10-11

**Authors:** R. W. Gilmour

**Affiliations:** St. Luke's Hospital


### REGISTERED HOSPITALS AND ASYLUMS.

By Dr. R. W. GILMOUR, M.B., B.S., St. Luke's Hospital.

The word " asylum " originally meant a place free from assault, a sanctuary, and thus a place of refuge for the helpless. It came to be used in the last sense in reference to the physically incapacitated—*e.g.*, blind asylum, deaf and dumb asylum, etc., as a rule denoting a place of retreat for those whose incapacity was permanent. The word " hospital," on the other hand, which originally indicated the entertainment of a stranger or guest, came to be used in connection with the sick as a place for the care and treatment of cases of a more temporary character. The connotations of the terms " asylum " and " hospital " still obtain, although sentimental reasons seem nowadays to tend to abolish the word " asylum " and to substitute " hospital " in the description of any institution for the sick. Especially is there a desire to abolish the opprobrious term and substitute the other one in the description of places for the treatment of mental diseases, and this is a perfectly natural desire on the part of anyone who is interested in the subject, either medically or from the point of view of educating the public.

In connection with the care and treatment of the insane the words asylum and hospital are technically defined in the Lunacy Act. An asylum is here defined as being " an asylum for lunatics provided by a county or borough, or by a union of counties or boroughs " ; whilst a hospital is defined as any hospital or part of a hospital or other house or institution (not being an asylum) wherein lunatics are received and supported wholly or partly by voluntary contributions, or by any charitable bequest or gift, or by applying the excess of payments of some patients for or towards the support, provision, or benefit of other patients."

Modern ideas of treatment of mental diseases insist more and more on the strict classification of patients and the separation of recoverable from chronic cases. The modern county asylum has an acute or hospital block separated from, though adjacent to, the rest of the institution, and attempts to drop the title asylum and to adopt that of county or borough " mental hospital." These two changes can but make for good, as by the one the acute cases are enabled to receive more individual attention than when they are mingled in wards with many chronic cases, and by the other the fear inspired by the word asylum is to a large extent removed from the minds of those who go to them. The segregation of the chronic insane and the treatment of the recoverable in separate institutions is, for financial and other reasons, not possible at the present time; nor does it seem likely to be so until all these institutions come to be under the control of the State, instead of being under that of the various counties and boroughs.

The " registered hospitals " number fourteen and are scattered over the country. They are all philanthropic institutions and are analogous to the voluntary general hospitals for physical diseases, being administered financially by committees appointed by the governors and subscribers. The majority of them possess only small endowments, and contributions towards the cost of maintenance have to be asked from the friends of patients to the extent that their circumstances permit. It must be remembered in this connection that owing to the average length of residence per patient and to certain expenses in dealing with mental cases which do not occur in the care of the physically sick, the cost of maintenance per head is above that of the general hospitals. The patients dealt with are those of the middle and upper middle classes who cannot afford to pay fees of the private institutions. The regulations for the admission of patients differ in the various registered hospitals, but most of them are supposed to be for the treatment of acute and recoverable cases. In all of them are to be found a certain number of chronic cases—selected carefully—but in those which carry out much charitable work and live up to the " hospital " ideal the number of these cases is kept as small as possible. The extent to which a registered hospital lives up to its name or sinks to the level of an asylum for chronic cases can be judged from the percentage of its annual admissions to its average number of patients resident. In some this percentage is commendably high, but unfortunately it must be owned that in others it is unbecomingly small. The amount of charitable work done seems also to vary very much, and, indeed, it would seem that in some of them the accumulation of property appears to be rather the primary consideration and not the full use of income for charitable aid.

The Commissioners in Lunacy constantly express regret in their reports that there are not more of these institutions and note the fact that the amount of charity done varies greatly among them. That they are needed is obvious from the fact that some counties have built institutions, or set aside parts of existing ones, for private cases for whose maintenance the friends can afford to pay a moderate sum. The registered hospitals rarely appeal to the public for funds, and their existence, the good work they do, and the great need there is for them are not generally realised.

For anyone interested in hospitals it is a most instructive study to scrutinise the statistics and financial statements of these registered hospitals and to note what great variations they show; to compare the ratios in them of floating and fixed population, representing acute and chronic cases, the recovery rates, and the cost of maintenance. A financial table giving the various items of income and expenditure in each hospital used to be published in the Annual Report of the Commissioners of Lunacy and afforded much interesting information, but the publication of this table was discontinued a few years ago. " Comparisons are odious " even in these enlightened days—at all events in hospital financial matters—and may be resented.

